# Electricity in the Diseases of Women, with Special Reference to the Application of Strong Currents

**Published:** 1889-08

**Authors:** 


### Electricity in the Diseases of Women, with Special Reference to the Application of Strong Currents, by G. Betton Massey, M. D., Philadelphia. F. A. Davis, Publisher, 1231 Filbert Street, Philadelphia. Price, $1.50.

This little book, of two hundred pages, is one of the most clearly-written works of its kind that we have seen. It is a plain exposition of the proper method of using strong electrical currents in the treatment of uterine diseases from functional dysmenorrhœa, amenorrhœa, etc., to fibroid tumors. It is mainly a record of the author's personal experience, and as he is a young man in the profession, it is a highly creditable record. He is also the inventor of an ingenious "current controller," illustrated on page 13 of his book. It consists of a disk of glass, covered over with a "tapering area" (suggestive of the curved arm of a crank) of plumbago. Over this plays a rotating lever, which, as it advances from the tapering end or point, covers more and more of the plumbago area, thus gradually increasing the electrical current. The book is well supplied with illustrations, and all the apparatus clearly explained.

Practical experiments are suggested to the beginner for learning the effects of electricity under varying conditions, by testing the current upon raw beef. These are certainly very desirable for gaining a clear insight into this method of treatment. Altogether it is a very clever book. W. M. J.

